# Artificial Modulation of the Gating Behavior of a K^+^ Channel in a KvAP-DNA Chimera

**DOI:** 10.1371/journal.pone.0018598

**Published:** 2011-04-19

**Authors:** Andrew Wang, Giovanni Zocchi

**Affiliations:** Department of Physics and Astronomy, University of California Los Angeles, Los Angeles, California, United States of America; German Cancer Research Center, Germany

## Abstract

We present experiments where the gating behavior of a voltage-gated ion channel is modulated by artificial ligand binding. We construct a channel-DNA chimera with the KvAP potassium channel reconstituted in an artificial membrane. The channel is functional and the single channel ion conductivity unperturbed by the presence of the DNA. However, the channel opening probability vs. bias voltage, i.e., the gating, can be shifted considerably by the electrostatic force between the charges on the DNA and the voltage sensing domain of the protein. Different hybridization states of the chimera DNA thus lead to different response curves of the channel.

## Introduction

Artificially controlled ion channels, whether by ligand binding [1], light [2], or other means, could form a useful research tool in neurobiology, as well as help understand the complexity of the functioning of natural channels. For instance, the recent artificial construction of a light-activated channel [3], and the cloning and expression of the rhodopsin channel in mice [4] stand to advance the field of neuroscience by providing local, non-invasive control over nerve cell stimulation. There are, of course, many ion channel blockers [5], [6] which have been used in medicine and research for a long time, but the question of modulating ion channel response is yet different. In this spirit, we are interested in ion channel-DNA chimeras, with an eye to eventually establish mechanical control over these molecules similarly to what we have demonstrated previously with enzymes [7]–[9]. Here we present observations obtained with KvAP-DNA chimeras, where however the DNA is not used as a mechanical spring [10], but rather as the provider of chemically controllable electrostatic forces.

KvAP is the voltage gated potassium channel from Archaea *Aeropyrum pernix* [11]. It is a tertramer consisting of four voltage sensing domains (VSD) surrounding a pore domain at the center. The positively-charged arginines in the fourth transmembrane helix S4 of VSD are the sensor for the membrane potential and they drive the helix S4 to move upon membrane depolarization. The conformational change of VSD is then transduced to the gate formed by the intracellular side of the S6 helix in the pore domain. To open the gate, it is believed that the four structurally-separated VSDs undergo a concerted conformational change, where the motion of each sensor is strongly coupled with one another through the S4-S5 linker and the pore [12].

**Figure 1:**
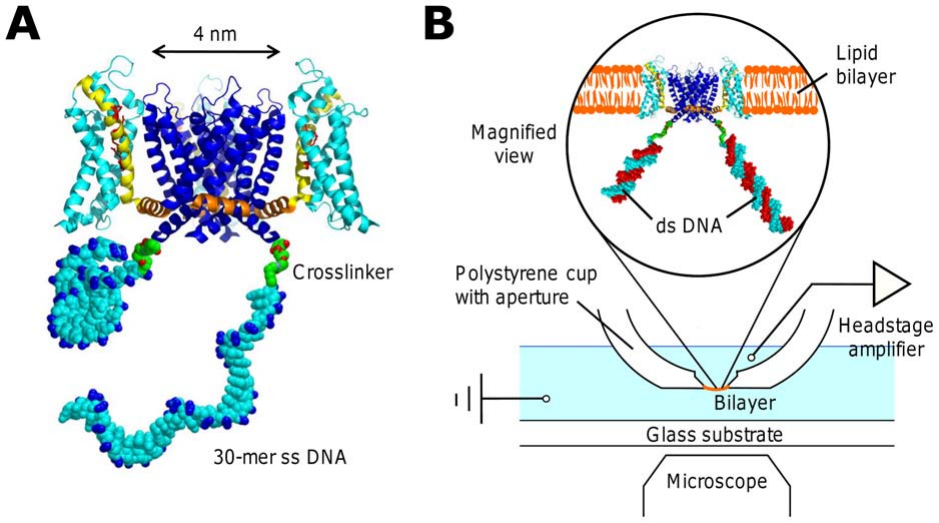
Cartoon of the KvAP-DNA chimera and the planar lipid bilayer setup. (A) The ssDNA strands are attached to the ends of the S6 helix. For simplicity, only two DNA strands in different configurational states are shown. The actual chimera has four DNA strands attached to the ion channel tetramer. The arginines on the S4 helix (yellow) are labeled in red and the phosphate groups in the DNA backbone are labeled in blue. The structure is prepared in PyMol and RasTop. PDB ID: 2A79. (B) Schematics of the setup showing that the chimera in the magnified view is hybridized to the complementary DNA strands in red.

Here we synthesize KvAP-DNA chimeras where a single strand of DNA is attached by one end to the S6 Helix of the monomer, producing reconstituted channels decorated with 4 strands of DNA (Fig. 1 A). The DNA produces a region of negative charge density in proximity to the four positively charged arginines on Helix S4 of the voltage sensor domain; the corresponding attractive electrostatic force pulls on the voltage sensor, biasing the channel towards the closed state. Hybridizing the chimera DNA shifts the charge density, decreasing the attractive interaction with the voltage sensor thus restoring the normal opening probability. The chimera channel is otherwise functional, in particular the single channel conductance in the open state is not affected by the presence of the DNA.

## Results

### The reconstituted KvAP-DNA chimera is functionally active

**Figure 2:**
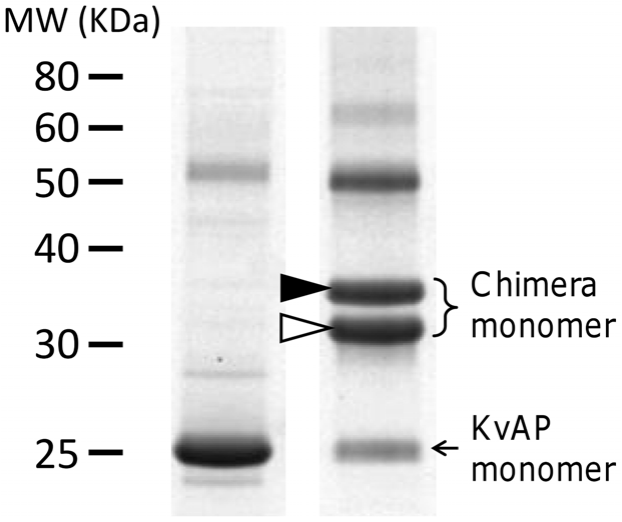
Protein expression and chimera synthesis confirmed by SDS-PAGE. The left lane is the V240C mutant protein and the right lane is the V240C chimera. The mol wt standard is shown on the left. The high mol wt bands in the chimera lane are KvAP-DNA chimera monomers. The open arrowhead indicates the monomer coupled with a Short B (SB) strand and the solid arrowhead the monomer coupled with a Long A (LA) strand. In SDS-PAGE, KvAP tetramers dissociate completely into monomers which migrate faster than expected with a mobility similar to a 20 kDa protein. The increased mobility is likely due to the higher tendency of membrane proteins to bind to SDS molecules [31]. After attached to chimera DNAs, two extra bands appear in the higher mol wt region showing the two products of conjugation.

The KvAP-DNA chimera is constructed by covalently coupling the amino-modified end of a DNA strand to the Cys substituted site 240 on the monomer, using a 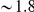 nm long crosslinker. The single-stranded DNA (ssDNA) strand is either Long A (LA, 30 mer), Long B (LB, 30 mer) or Short B (SB, 20 mer)(Table 1). Two chimeras are constructed separately: one with LA and LB strands (1∶1), the other with LA and SB strands (1∶1). In either case the conjugation yield is high (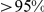, Fig. 2). The reconstituted tetrameric channels are therefore mostly decorated with 4 DNA strands, in various combinations of LA and LB or SB. For the mutant V240C we obtained a high coupling efficiency to the DNA, possibly because this attachment site is highly exposed and distant from lipid/detergent bound regions (Fig. 1 A). This DNA attachment site is located at the intracellular end of the S6 helix, where the gate of the channel resides.

**Table 1:** DNA sequences used in chimera synthesis.

Name	Length (bases)	Sequence (5′ 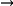 3′)
Long A	30	GAGTGTGGAGCCTAGACCGTGAGTTGCTGG
Long B	30	CAGGAGTCCACGGTACCATCCAAGCAGCTG
Short B	20	CAGGAGTCCACGGTACCATC

After channel reconstitution, we had both orientations of the channel in the vesicle bilayer, which resulted in some channels being inserted into the suspended bilayer with the “correct” orientation, i.e., the intracellular (DNA decorated) side facing the *Cis* (upper) chamber, while some channels were oriented reversely, with the DNA decorated side facing the *Trans* chamber. When holding the membrane voltage 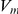 at 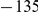 mV (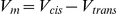), the correctly oriented channels are held closed while the oppositely oriented channels inactivate after prolonged opening.

**Figure 3:**
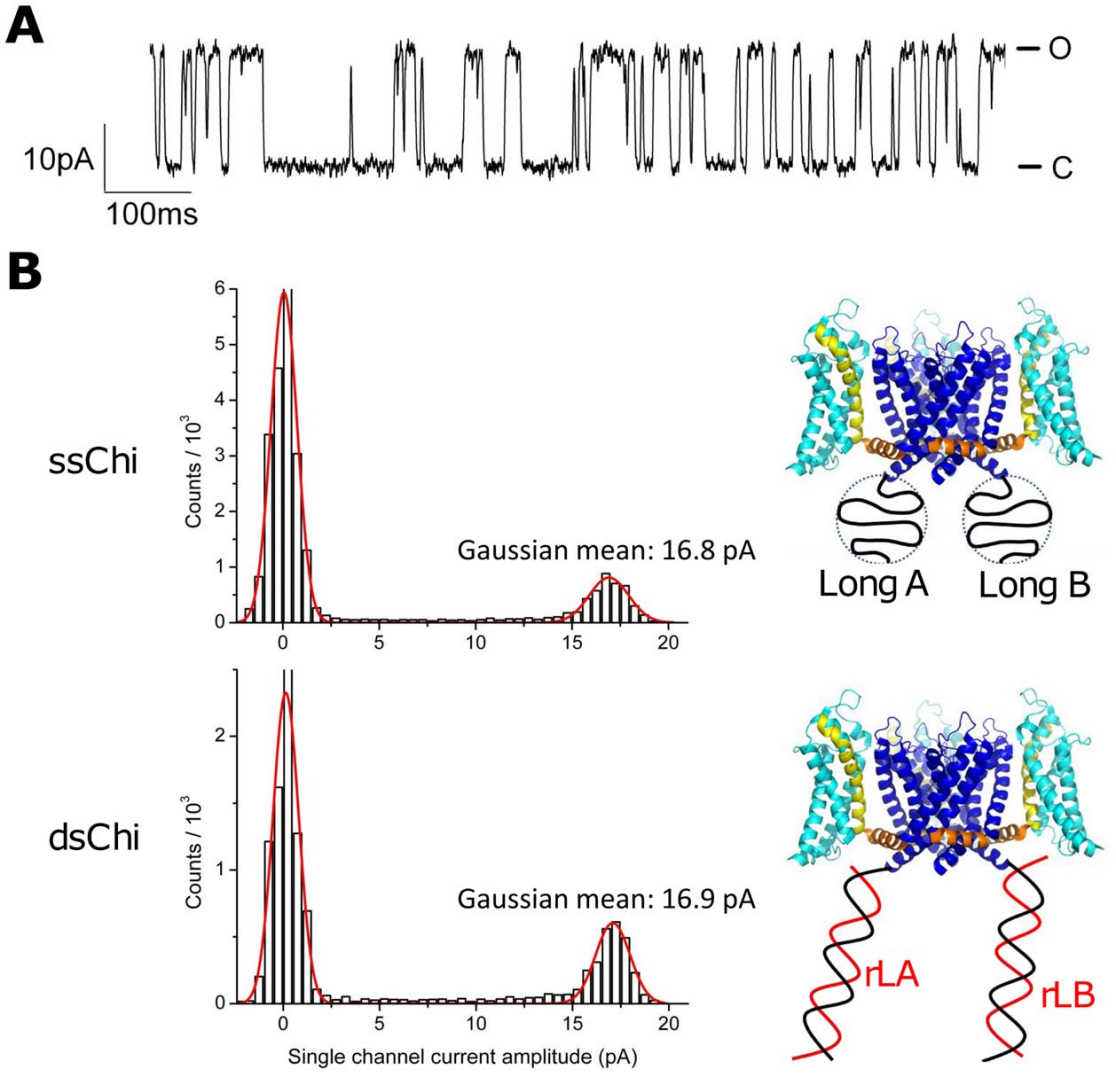
Single channel current of the KvAP-DNA chimera. (A) Single channel recording of ssChi with the membrane depolarized to +95 mV. The upper state is the open (O) state and the lower state is the close (C) state. (B) Histograms of the single channel current before (ssChi) and after (dsChi) hybridizing to the direct complements rLA and rLB. At 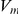 = +95 mV, the conductance in both cases was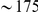 pS. Channel configurations are shown on the right. The chimera DNA strands are in black and the complements are in red.

Single channel activity was sometimes seen in the late part of the multi-channel recordings after most channels were inactivated (Fig. 3). We measured the single channel current from these recordings, and found a conductance (I/V) of 175 pS (Fig. 3 B), essentially the same as the 170 pS conductance of the wild-type KvAP [11]. The function of the ion channel is thus preserved with DNA strands attached to each monomer subunit. Moreover, hybridization of the chimera DNAs to their complementary strands did not affect the single channel conductance (Fig. 3 B).

To summarize: the KvAP-DNA chimera is functional, and the single channel conductance unchanged by the presence of the DNA.

### ssDNA attached to the intra-cellular side of the channel inhibits the macroscopic current

**Figure 4:**
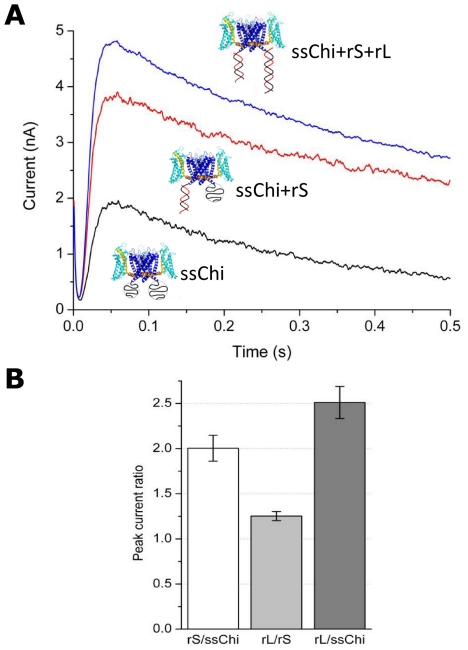
Macroscopic current (Multi-channel recording) of the KvAP-DNA chimera (LA+SB) in different hybridization states. (A) The current vs. time for three different hybridization states. The currents before (ssChi) and after (ssChi+rS or ssChi+rS+rL) hybridization with the complementary DNA were measured from the same preparation (measured in a sequence without membrane breakage or insertion of new channels into the bilayer). (B) Comparison of peak currents in different hybridization states: rS/ssChi is the peak current ratio of ssChi+rS to ssChi in (A); rL/rS is the ratio of ssChi+rS+rL to ssChi+rS; and rL/ssChi is the overall effect between states ssChi+rS+rL and ssChi. The data are the average of three to five measurements; the error bars are 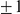 SD.

When 10–100 channels are present in the membrane one measures a macroscopic current (Fig. 4 A). Upon depolarization from the resting potential (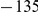 mV) to a positive test potential (+60 mV), a large current of the order of nA was observed. After the initial drop of the capacitive current, the macroscopic current increases rapidly due to the stochastic opening of the channels. The current reaches a maximal value (the peak current, 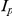) after 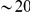 ms, corresponding to all channels being open. Then it decreases slowly exponentially with a time constant of 0.4–1 s. The peak current 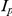 is well defined, owing to the separation of time scales between the fast rising (channels opening) and slow decrease (channels inactivating). The decrease of the current is due to the well known inactivation process of voltage-dependent ion channels [13]. For the data of Fig. 4, the KvAP-DNA chimera was built with the DNA strands LA and SB. The Figure shows the effect on 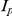 of manipulating the spatial charge distribution through DNA hybridization (recall that ssDNA of this length is a random coil, whereas double-stranded DNA (dsDNA) of the same length is a rigid cylinder.) Hybridization of SB (using the complementary rS, Table 2) leads to an increase of 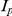 of a factor 2. Further hybridization of LA causes an additional increase by a factor 1.25. The total increase in 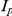 was therefore 2.5-fold when both SB and LA were hybridized (Fig. 4 B).

**Table 2:** Complementary DNA sequences used in hybridization experiments.

Name	Abbreviation	Hybridization targe	Length (bases)	Sequence (5′ 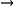 3′)
rLong A	rL or rLA	Long A	30	CCAGCAACTCACGGTCTAGGCTCCACACTC
rLong B	rLB	Long B	30	CAGCTGCTTGGATGGTACCGTGGACTCCTG
rShort B	rS	Short B	20	GATGGTACCGTGGACTCCTG
Distal Charge	DCH	Short B	50	GATGGTACCGTGGACTCCTGTCCGTCATGCATCTCCTCAGACTGATACTC
Proximal Charge	PCH	Short B	50	TCCGTCATGCATCTCCTCAGACTGATACTCGATGGTACCGTGGACTCCTG

We interpret these results as follows. With the DNA in the ss state, there is a cloud of negative charges close to the channel on the intra-cellular side. This inhibits the peak current in multi-channel recordings by as much as 2.5 fold. Hybridization of the chimera DNA shifts the negative charge distribution away from the channel, removing the inhibition of 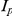. Since we know from the single-channel measurements that the chimera DNA does not affect the single channel conductance (Fig. 3 B), the large increase of peak current after hybridization must be due to a depressed opening probability in the ssChi state (The chimera before hybridizing to any complementary DNA is referred to in the following as the single-stranded chimera (ssChi)). This result suggests that electrostatic interactions between the chimera DNA and the voltage sensing domain of the protein modulate the channel opening probability, as discussed in detail below.

### The DNA charge affects the gating behavior

DNA is a polyelectrolyte with one negative charge per nucleotide. Single-stranded DNA has a persistence length of 3 bases, thus a ssDNA 30-mer is a flexible chain with a random coil size of about 3 nm. The chimera DNA therefore produces a mobile negative charge distribution in close proximity to the intra-cellular side of the voltage sensing domain (Fig. 1); the DNA chain has the necessary degrees of freedom to place, on average, 2–3 nucleotides positioned as close to the positively charged Arg on the S4 Helix of the voltage sensing domain as steric constraints permit. The electrostatic interaction energy of one negative charge with these arginines is at least 1 kT (see later). Thus the minimum free energy configuration will be one in which the charge distribution of the DNA is somewhat concentrated at the points of closest approach to the Arg of Helix S4. On the other hand, the charge distribution of the 30-bp dsDNA is constrained on a 10 nm long rigid rod, which merely lingers more often around the interface as described in [14]. Note that the electrostatic energy is dominated by the closest charge pair, as the fields fall off exponentially in the solvent with a Debye length of 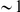 nm. Therefore, although dsDNA has twice the charge of ssDNA, most of this charge is far beyond the Debye length from the gate arginines. ssDNA on the other hand can adjust its conformation to pack more charges within the effective peripheral of the gate charges. It has been shown both by experiments and simulations [15], [16] that the S4 arginines are not always embedded in lipids. In the closed state, the arginines are accessible from the intracellular side and could directly contact the flexible ssDNA. Double-stranded DNA, on the other hand, due to its rigid conformation could only have limited interactions with the S4 arginines through such direct contacts.

**Figure 5:**
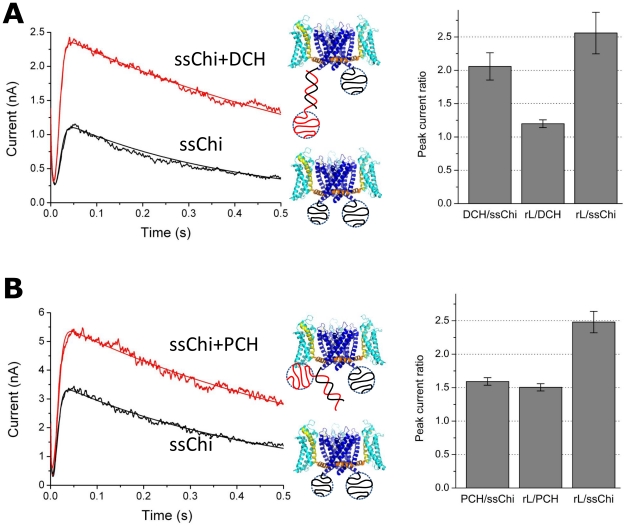
Manipulating the DNA spatial charge distribution by hybridizing to different strands. Hybridization of DCH adds a blob of 30-mer ssDNA to the end of the dsDNA which is away from the channel, while PCH adds the same 30-mer blob in the close proximity to the intracellular side of the channel. (A) Macroscopic current before and after hybridization of DCH. Comparison of peak currents in three different hybridization states (ssChi, ssChi+DCH, and ssChi+DCH+rL) is shown in the plot on the right. The ratios are labeled in the same way as in Fig. 4 B. (B) Macroscopic current before and after hybridization of PCH and the peak current ratios between different hybridization states. The data are the average of three to five measurements; the error bars are 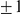 SD. The curves on top of data were from the fitting to a formula: 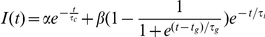 including the initial exponential decay of the capacitive current (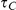), the sigmoid growth of the rising current (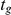 and 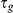) and a slow exponential decay due to channel inactivation (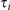).

In order to probe these qualitative ideas we performed a series of experiments where we manipulate the DNA charge distribution. An extra piece of 30-mer ssDNA was added to either the 5′ or the 3′ end of rS, the complementary to the chimera DNA SB (Distal charge (DCH) and Proximal Charge (PCH), respectively; see Table 2). Hybridization with DCH adds a blob of 30-mer ssDNA to the distal end of the dsDNA while PCH positions the ssDNA blob close to the voltage sensor (Fig. 5).

As shown in Fig. 5 A, hybridization of DCH resulted in a 2-fold increase of peak currents similar to the result obtained with the original complementary DNA rS. In contrast, hybridization with PCH, which adds the same blob of 30-mer ssDNA right below the protein (Fig. 5 B), resulted in a 1.6 fold increase in current, smaller than the 2-fold increase in the case of DCH (or rS). After adding rL, a 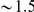 fold increase occurred and brought the overall effect to the same 2.5 fold increase with respect to the ssChi state (Fig. 5 B, right). Given the same number of net DNA charges in the system and the same maximal effect, the differential responses in these hybridization experiments are reasonably accounted for by the different charge distributions. The ssDNA attracts the voltage sensing arginines electrostatically and biases the channel towards the closed states. After hybridization, the electrostatic attraction between dsDNA and the arginines is weakened and the voltage sensing domain is set free.

### Estimate of the electrostatic attraction between the DNA charges and the S4 Helix

The main contribution of the electrostatic potential energy between charges on the DNA strand and the S4 helix comes from the closest pair of DNA and arginine charges. Determined from the solved crystal structure of KvAP voltage sensor in the open state of the channel [17], the distance of closest approach is 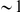 nm. Water, with its large dielectric constant, attenuates the field of DNA charges so that the effective DNA charge seen by the arginine is reduced by a factor 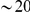 (
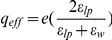
, where 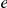 is electron charge, 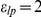 the dielectric constant of lipid or protein, and 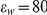 the dielectric constant of water [18]). This gives an electrostatic energy of 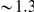 kT between the closest charge pair. This is actually a lower bound, resulting from using the dielectric constant of bulk water in the estimate. The complex environment of the interface containing the hydrophilic surface of the channel protein and the polar headgroup of the zwitterionic lipid DPhPC can result in a smaller effective dielectric constant [19] and correspondingly larger energy. In addition, this energy becomes even larger when the channel is in the closed state with the arginines translocated to the level of the lipid-water interface.

### Gating response for different hybridization states of the chimera

**Figure 6:**
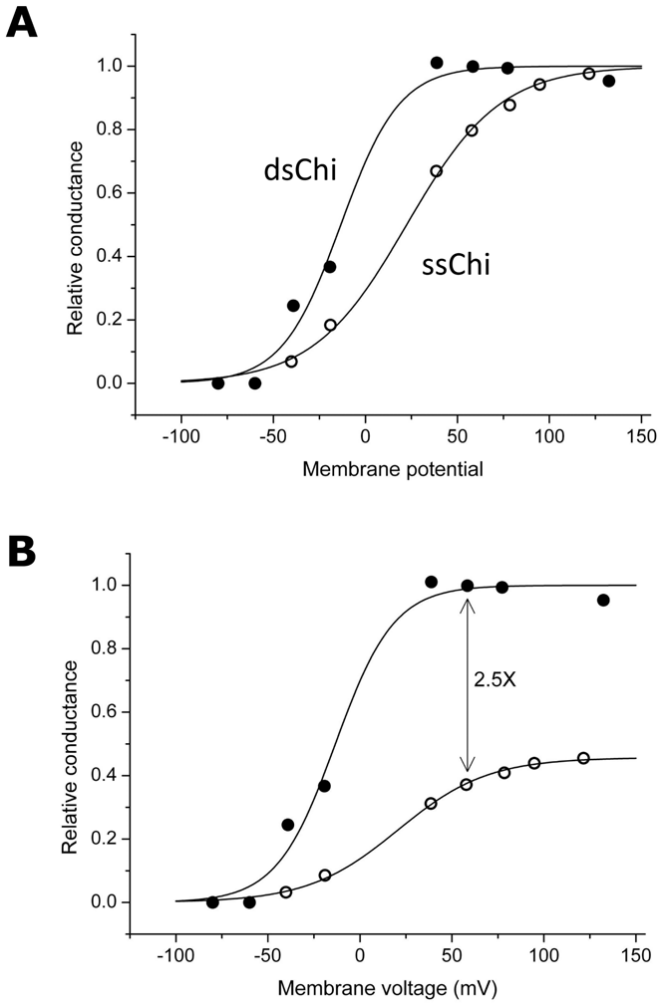
The gating response (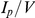 vs. 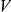 curves) of the KvAP-DNA chimera in the unhybridized and hybridized states. The conductance is measured from the peak current 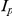 in multi-channel recordings, and normalized to represent the opening probability of the channel. (A) The conductance of ssChi (open circle) and dsChi (ssChi+rS+rL, close circle), with the maximum conductance normalized to 1 in both cases. (B) The same data in (A) but the maximum conductance of ssChi was re-normalized to exhibit a 2.5-fold inhibition as measured in Fig. 4. The curves on top of the data are from fitting to a sigmoid growth function.

Fig. 6 shows the voltage dependence of the apparent conductance (I/V vs. V curve, also known as the G/V curve, where I is the macroscopic current) of the KvAP-DNA chimera in the ss and fully hybridized states. The conductance was calculated from the peak current (see Fig. 4) at different test potentials divided by the corresponding voltage. When both strands were hybridized to their complements (rS+rL), the G/V curve obtained was similar to that of the wild-type KvAP in DPhPC lipids [13]. The G/V curve of ssChi shows a shifted half activation potential to a more positive voltage and also a less sharp voltage response.

If we normalize these G/V curves to the largest conductance in each curve, the shift between the two curves at half activation is 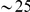 mV (Fig. 6 A). However, we know from Fig. 4 that there should be a factor of 2.5 difference between the peak current for the two states at +60 mV. If we therefore normalize the G/V curve for ssChi as shown in Fig. 6 B, we find that the conductance of ssChi never reaches the level of the original channel, even at +120 mV. In short, by hybridizing the chimera DNA we are able to substantially alter the gating response of the channel.

## Discussion

Attaching ssDNA to the ion channel substantially alters the gating behavior of the channel as seen in the positive shift the the G/V curves with respect to the untagged wildtype KvAP channel (Fig. 6). On the other hand, the single channel conductance remains unchanged with either ss or ds DNA attached to the channel (Fig. 3). Thus the DNA couples to the dynamic gating process but not to the static current conduction, indicating that an electrostatic interaction between the DNA charge and voltage sensors is affecting the conformational transition associated with channel gating.

The changes in the macroscopic peak current 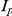 observed when manipulating the charge distribution due to the DNA are accounted for by the change in the opening probability of single active channels. Two different processes – voltage gating and inactivation – determine the number of open channels. However, because of the large separation of time scales between the rise of the macroscopic current (Fig. 3) due to stochastic channel opening after depolarization (characteristic time scale 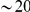 ms) and the slow drop of the current due to channel inactivation (characteristic time scale 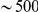 ms) the changes observed in the peak current must be due to changes in the opening probability of active channels only.

In addition, we do observe changes to the time scale for inactivation occasioned by manipulating the DNA charge. Namely, we observe that the KvAP-DNA chimera inactivates faster (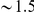
–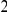 fold) in the ss state than in the hybridized state. We do not have yet an insightful picture of this effect, so we do not discuss it at length. We merely note that, as is well known, with prolonged opening Kv channels slowly become non-conductive, entering the so-called inactive state. Recent work has addressed the voltage dependence, the influence of lipids, and detailed interactions between the filter and its surrounding structure in the context of Kv channel inactivation [13], [20]. The time scale of inactivation depends on the concentration of K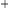 on both sides of the membrane. The higher the concentrations, the slower the channel inactivates [21]. This fact was also confirmed by the structure study of a potassium channel in low salt crystallization conditions, showing a constricted filter conformation [22]. The reason for inactivation has to do with the occupancy of the filter; ions inside the filter stabilize it against collapse. Lowering the ion concentration dilutes the average number of ions in the filter and results in faster inactivation. Thus inactivation is not independent of the opening probability, which could be the reason why we see changes in the inactivation time scale in our experiments, where we modulate the opening probability.

In conclusion, we have constructed KvAP-DNA chimeras where the charge on the DNA, which can be manipulated externally by hybridization, pulls electrostatically on the voltage sensing domain, biasing the opening probability of the channel. The resulting gating response (the opening probability vs. bias voltage, Fig. 6) is substantially modified, whereas the single channel conductance is unperturbed.

It is of course not surprising that one can bias a voltage gated channel through electrostatic interactions, and indeed phosphorylation is used by the cell to that effect [23], [24]. The details are however not simple, for instance, phosphorylation of the Kv channel on giant squid axons (intra-cellular side) has recently been shown to shift the gating behavior, but in the opposite direction to that found here [25]. The present *in vitro* system may be a well controlled tool to study these effects in detail. For potential applications, this artificially-controlled channel could be delivered to living cells such as neurons possibly through endocytosis of liposomes with reconstituted artificial channels. By designing the addressable sequence of the ssDNA to bind with specific targets, the artificial channels embedded in cells could be made to couple with parts of the regulatory pathways such as micro RNAs or an aptamer-binding protein.

## Materials and Methods

### Mutagenesis of KvAP gene

The cloned gene of the voltage-dependent K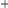 channel from *Aeropyrum pernix* (KvAP, a gift from Prof. MacKinnon, The Rockefeller University) was modified by site-directed mutagenesis (QuikChange, Stratagene, CA) to remove the single native cysteine at the sequence site 247 (C247S) and to add a single cysteine mutation at the sequence site 240 (V240C). The mutants were confirmed by gene sequencing performed by UCLA GenoSeq Laboratory. The mutant KvAP protein was expressed in *E. coli*.

### Expression and purification of KvAP protein

All the procedures in the following sections were performed at room temperature. The procedure of expressing KvAP was adapted from published procedures [11]. The protein expressed in 10 g of *E. coli* resuspended in 50 mL Lysis Buffer (50 mM Tris at pH 8.0, 100 mM KCl, 0.2 mg/mL lysozyme, 2 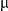
g/mL DNase, 2 mM 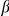-ME and protease inhibitor cocktail (Sigma, St. Louis, MO)) was harvested using French Press. The protein was extracted from cell lysates with 40 mM detergents decylmaltoside (DM, Abatrace, Maumee, OH) for 3 h, cleared by centrifugation, and purified on a Talon affinity column (Clontech, Mountain View, CA).

Before adding to the protein supernatant, the Talon beads were washed with 30 mL Wash Buffer (20 mM Tris at pH 8.0, 100 mM KCl, 10 mM imidazole, and 5 mM DM). The bead-supernatant mixture was gently rotated for 1 hour and passed through the column 4 times. Non-specifically bound protein was removed by washing the beads thoroughly with 30 mL Wash Buffer, and the channel protein was eluted with 8–10 mL Elution Buffer (same as Wash Buffer, except for the additional 400 mM imidazole). 1.5 units of thrombin were added to each mg of eluted protein right after elution to remove the his-tag overnight. The protein treated with thrombin was reduced by 5.0 mM TCEP for 30 min to regenerate free sulfhydryl groups and then run on a size-exclusion Superdex-200 column (GE Healthcare, Piscataway, NJ) in HPLC Buffer (20 mM Tris at pH 7.5, 100 mM KCl, and 5.0 mM DM).

### KvAP-DNA chimera synthesis

Custom-made amino-modified DNA strands (Integrated DNA Technologies, Coralville, IA) with different sequences and lengths were used to attach to the ion channels (Table 1). The sequences were randomly selected with the requirement of minimized secondary structures (i.e., with a hairpin length shorter than 3 bp). The ssDNA thus behaves as a random coil. These strands are attached to the channel in one single step and can result in KvAP-DNA chimera molecules with different number of attached DNA strands. For each nmole of channel protein, 6 nmols of each DNA strands were used to give a DNA/cysteine molar ratio of 3∶1. The two DNA oligos were first mixed (1∶1 molar ratio) and incubated with 50 times of NHS-PEO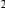-Maleimide (Pierce, Rockford, IL), in Conjugation Buffer (100 mM sodium phosphate, 150 mM NaCl, and 1 mM EDTA at pH 7.5) for 1 h. The DNA-crosslinker construct was then passed through the Superdex-200 column in HPLC Buffer to remove excess uncoupled crosslinkers. Corresponding DNA-crosslinker fractions were collected and concentrated to 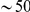 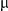
M. The protein treated with thrombin was immediately added to the DNA-crosslinker construct after purified by HPLC. The DNA-crosslinker-protein mixture was allowed to react overnight and purified later on the Superdex-200 column to remove uncoupled DNA oligos. The final KvAP-DNA chimera was concentrated to 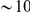 mg/mL and confirmed by SDS-PAGE with SYPRO Ruby protein staining (Invitrogen, Carlsbad, CA).

### Reconstitution of KvAP-DNA chimeras

Reconstitution of KvAP-DNA chimeras into lipid vesicles was adapted from the method in Heginbitham *et al.* [26]. DPhPC lipids (Avanti, Alabaster, AL) in chloroform were dried with a gentle blow of nitrogen gas in a glass tube, washed with 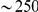 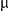
L pentane and then placed under room vacuum for 30 min. The dried lipids were resuspended in Reconstitution Buffer (10 mM HEPES-KOH at pH 7.4 and 450 mM) at a lipid concentration of 20 mg/mL by vigorously vortexing for 30 min. The lipid solution was then sonicated at room temperature for 20–30 min to form unilamellar vesicles. The vesicles were dissolved in 10 mM DM for 30 min and the KvAP-DNA chimera was incorporated into the lipid/detergent mixture to a chimera/lipid ratio of 0.1–0.2 (wt/wt). The detergent concentration was then increased to 17.5 mM and the mixture was incubated for 2 h with gentle vortexing every 20 min.

The reconstituted chimera vesicles was formed by removing DM from the mixture using spin-desalting columns (Pierce) and detergent absorbents Bio-Beads (Bio-Rad, Hercules, CA) [27], [28]. The mixture was first passed through the desalting column 3 times and then transferred to a tube with sufficient Bio-Beads, which were washed with methanol, deionized water, and Reconstitution Buffer before use. The mixture solution was transferred to fresh beads every 12 h. After 4 rounds or 48 h, the detergent-free chimera/lipid vesicles were divided into aliquots, flash frozen using a dry ice and ethanol bath, and stored away at 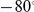
C.

### Electrophysiological measurements with a planar bilayer setup

Planar lipid bilayers were used to measure single channel and macroscopic currents. We used support apertures 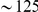 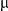
m in diameters. Following the melt-and-shave method described in Wonderlin *et al.* [29], a cone-shaped dent was made by pressing a coil-heated sharp steel stylus into the inner surface of a plastic Ultra-Clear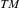 centrifuge tube (Beckman Coulter, Fullerton, CA) and the tip of the cup was then shaved off to reveal the aperture. The platform is assembled on a standard inverted microscope for viewing bilayer formation optically (Fig. 1 B).

DPhPC lipids were freshly prepared each time following the same procedure mentioned earlier. Dried lipids were then dissolved in n-decane to a concentration of 20 mg/mL. For a stable bilayer to form, first a droplet of lipids (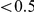 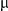
L) was applied to the aperture and allowed to dry in air. A second droplet was then added to the aperture and before it dried out, both chambers were filled with *Cis* Buffer (10 mM HEPES-KOH at pH 7.4 and 150 mM KCl) to the same height to avoid a hydraulic pressure difference across the bilayer. The planar lipid bilayer was then painted over the aperture using a miniature glass spatula. The bilayer forms spontaneously when excess lipids are removed by the spatula.

To insert the chimera into the bilayer, a fresh aliquot of KvAP-chimera vesicles was removed from the freezer and thawed at room temperature. The vesicle solution was then gently vortexed and bath-sonicated for 2
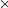
5 seconds. A concentrated dose of vesicles (1 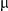
L) was pipetted on top of the bilayer and the current at a 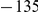 mV holding voltage was monitored. Usually the vesicle can fuse in a few minutes, possibly due to the swelling of vesicles (since the potassium concentration in vesicles is three times higher than that in the bathing buffer [30].) Once the channels were inserted in the bilayer as revealed by the current, the concentrated vesicles in the vicinity of the bilayer were diluted down by stirring and mixing the solution in the *Cis* chamber.

Macroscopic and single channel currents were detected using a home-built voltage-clamp amplifier with a 20 M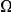 feedback resistor and further amplified and filtered with a low-noise preamplifier (SR560, Stanford Research System, Sunnyvale, CA). The signal was then digitized using an analog-to-digital converter (NI USB-6009, National Instrument, Austin, TX) and recorded using the software NI LabVIEW Signal Express (National Instrument). A battery-powered pulse generating circuit was used to apply the voltage across the membrane. Typical holding voltages were 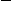
135 mV and channel currents were measured after stepping to different test voltages ranging from 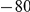 mV to +200 mV for various time lengths from 0.1 to 10 s. The currents were filtered at 0.3–1 kHz and sampled at 1–4 kHz.

### Hybridization experiments

The chimera DNA strands attached to the channel were hybridized to different complementary DNAs to change the conformation or the charge distribution of the DNA close to the voltage sensor of KvAP. The complementary strands can be: (a) simple, direct complements of the chimera DNA arms (rLong and rShort); or (b) complements with an additional decoration of an extra 30 bases attached at the 5′ or the 3′ end (Proximal Charge (PCH) and Distal Charge (DCH), respectively; see Fig. 5). A list of these DNA sequences is given in Table 2.

Once inserted in the membrane, the ssChi was depolarized to the test potential and the currents were repeatedly measured for at least five times or until no obvious changes were observed. Each depolarization test was separated by at least 15 seconds for the channel to recover from the inactivation state. To initialize hybridization, the complementary DNA was pipetted to the *Cis* side of the membrane to a final concentration of 0.5–1 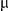
M (According to the electrophysiology convention, the *Cis* chamber is the intracellular side of the cell. Since the chimera DNAs were attached to the intracellular side of the channel, the complementary DNA is added to the *Cis* chamber.) The complementary DNA was left unperturbed to diffuse and hybridize to the chimera DNA for at least 2 min. The hybridized chimera channel was then depolarized following the same protocol of ssChi and the currents were measured repeatedly for 3–5 times. The same procedure was used for further hybridization with other complementary DNA.
